# Phosphorylated Neurofilament Heavy Chain Correlations to Visual Function, Optical Coherence Tomography, and Treatment

**DOI:** 10.1155/2010/542691

**Published:** 2011-03-03

**Authors:** Joshua Pasol, William Feuer, Cui Yang, Gerry Shaw, Randy Kardon, John Guy

**Affiliations:** ^1^Bascom Palmer Eye Institute, Miller School of Medicine, University of Miami, 1638 NW 10th Avenue, Miami, Fl 33136, USA; ^2^Department of Neuroscience, McKnight Brain Institute, College of Medicine, University of Florida, Gainesville, FL 32610-0015, USA; ^3^Department of Ophthalmology and Visual Sciences, the University of Iowa and Veterans Hospital, Iowa City, IA 52242, USA

## Abstract

*Objective*. To correlate visual and neurologic clinical scores and treatment of optic neuritis and multiple sclerosis (MS) patients with assays of serum phosphorylated neurofilament heavy chain (pNF-H) and optical coherence tomography (OCT) measurements of axonal loss. *Design/Methods*. The Optic Neuritis Treatment Trial (ONTT) randomized 457 patients with acute optic neuritis to intravenous methylprednisolone (IVMP) followed by oral prednisone, oral prednisone or placebo treatment arms. We quantified serum pNF-H levels in 175 ONTT patients 5 years after study entry. We performed OCT measurements of macular volume and the retinal nerve fiber layer (RNFL) in a subset of 51 patients at year 15. *Results*. Elevated pNF-H levels at year 5 correlated to poorer visual function at study entry. Lower 15 year macular volumes and RNFL thickness correlated better with follow-up than with baseline visual function measures. With IVMP treatment, 15 year RNFL differences of the fellow eye (FE) minus the affected eye (SE) RNFLFEmSE correlated with five-year pNF-H levels. PNF-H was reduced by half with IVMP relative to placebo or by 40% relative to prednisone. *Conclusions/Relevance*. Acute optic neuritis patients who have more severe visual loss during initial presentation have a higher incidence of axonal loss that was slightly suppressed with IVMP treatment.

## 1. Introduction

The Optic Neuritis Treatment Trial (ONTT) compared three modes of treatment for acute unilateral optic neuritis: placebo, oral prednisone, or high-dose IVMP followed by oral prednisone [1]. Enrollment of 457 patients by 15 centers started in 1988 and was completed 3 years later. Results of the study showed that visual acuity recovered 2 weeks faster with IVMP. Six months after treatment, no difference in the generally good visual outcome was detected between the 3 treatment groups. Similarly, there were no differences in the number of a minority of patients with a poor visual outcome varying between 5% and 6% for each group and defined by the study as 20/50 or worse. 

Axonal and neuronal loss are increasingly recognized as the primary factors contributing to persistent deficits and disability in multiple sclerosis (MS) and optic neuritis [2–4], as also revealed by optical coherence tomography (OCT) [5, 6]. Neurofilaments are components of the axonal cytoskeleton that consists of several subunits, including a light, medium, and heavy chain (NF-L, NF-M and NF-H). They are released into the blood stream and CSF with axonal disruption [7] that is believed to be mediated by inflammatory cells in multiple sclerosis [8]. The heavily phosphorylated axonal form of NF-H, called pNF-H, is resistant to proteases and relatively easy to detect [9, 10]. Petzold and coworkers have described elevated levels of pNF-H during the acute phase of visual loss from optic neuritis [11–13]. NF-H levels correlated with poor visual recovery and the development of MS. We wondered whether serum pNF-H levels may be a useful gauge of axonal loss in a large cohort of optic neuritis and MS patients. 

## 2. Methods

### 2.1. Clinical Assessments

ONTT patients underwent visual acuity, visual field, and contrast sensitivity testing at study entry then at the one-month, 6-month, 5-year, 10-year, and 15-year follow-up visits. EDSS assessments were performed at the 5-year, 10-year, and 15-year follow-up examinations. MRI was performed at baseline and at the five-year examination [14–17]. 

### 2.2. pNF-H Assay

Serum derived from venous blood was drawn during follow-up examinations 5 years after patient enrollment into the treatment arm of the study. Serum was stored in a −80 freezer for 10 years. Serum was assayed for the presence of phosphorylated neurofilament heavy chain (pNF-H), using a recently described monoclonal antibody-based assay [18]. Wells of microtitre plates were coated overnight with 100 *μ*L of purified pNF-H monoclonal capture antibody clone AH1, diluted in 10 mL 0.05 M carbonate buffer, pH 9.5 to give a final concentration of 1 *μ*g IgG per ml. The antibody and carbonate mix was decanted and the plates blocked with 150 *μ*L of 5% nonfat milk in TBS for 1 hour. The plate was washed with 2% nonfat milk in TBS and 0.1% Tween 20 (pH 7.5). After washing, a total of 50 *μ*L of standard or 20 *μ*L serum sample plus 30 *μ*L 2% nonfat milk in TBS and 0.1% Tween 20 were added in duplicate to the plate. The plates are incubated on a shaker at room temperature for 1 h. After washing, 100 *μ*L of purified mouse anti-pNF-H monoclonal antibody, directly coupled to horse radish peroxidase (HRP) at a final concentration of 1 *μ*g/mL in 2% nonfat milk in TBS plus 0.1% Tween 20 were added to each well and the plate incubated for 1 h at RT. After a final wash, the reaction was visualized using 100 *μ*L per well of 3,3′,5,5′-tetramethybenzidine HRP developer solution (Thermo Fisher, Rockford, IL). Absorbance was read at a wavelength of 450 nm on a Tecan SpectraFluor ELISA plate reader, 15 minutes after addition of chromogen, and after stopping the reaction by the addition of 50 *μ*L 1M H_2_SO_4_ per well. The entire set of samples were run as described twice with excellent reproducibility. The pNF-H antibodies used here can be obtained commercially from EnCor Biotechnology Inc. (Gainesville, FL).

### 2.3. OCT

Optical coherence tomography (OCT) [19, 20], an indirect way of measuring retinal nerve fiber layer thickness around the optic nerve, and macular volumes were obtained in a subset of 51 patients, 10 years after drawing blood samples (15 years after ONTT enrollment). Peripapillary retinal nerve fiber layer (RNFL) thickness was evaluated using the fast RNFL program of the Stratus OCT (Carl Zeiss Meditec, Dublin, CA), and analyzed using software version 3.0. RNFL thickness is determined at 256 points around a circular scan (diameter 3.4 mm) around the center of the optic disc that is repeated 3 consecutive times. For each eye, RNFL scans were repeated 4 times, exported on an electronic worksheet, and an average scan was computed. RNFL thickness and macular volume were evaluated from the average scan. OCT data from the clinical centers was collected by the reading center at the University of Iowa.

### 2.4. Data Analysis

Pearson correlation analysis of data was performed using SPSS statistical software (Chicago, IL). The association of subject characteristics to pNF-H levels was studied with multiple regression. The pNF-H data were square root transformed to effect normality of distribution of residuals. Relationships between the square root of pNF-H were investigated within groups with Pearson correlation and the two-sample *t*-test. Linear regression was used to assess the difference between groups and whether the relationship was different between clinically isolated optic neuritis patients and those with clinically definite MS. Analysis of covariance was used to assess the influence of treatment on pNF-H levels.

## 3. Results

### 3.1. pNF-H and Treatment

**Figure 1:**
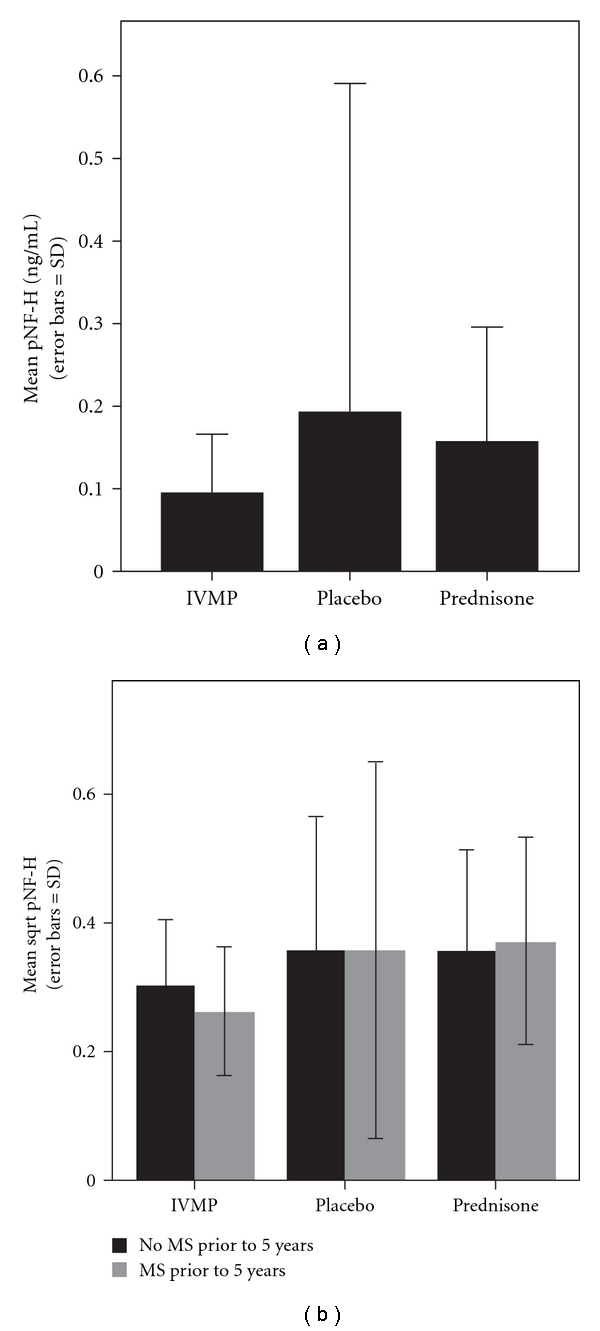
Bar plots of pNF-H concentration (nanograms per mL) in optic neuritis patients by treatment group (a) and between optic neuritis cases with or without MS at 5 years (b). Error bars = standard deviation.

We quantified pNF-H levels in the serum of 175 (44%) of the 397 patients, 87% of those initially entered into the ONTT, who returned for follow-up examinations 5 years after enrollment into the study. Their baseline characteristics are listed in Table 1. A normal control group of age-matched volunteers free of any neurologic diseases had a mean pNF-H concentration of 0.07 ± 0.05 nanograms (ng) per milliliter (mL) (mean ± standard deviation) (*n* = 12). Sixty-three of the ONTT patients had received IVMP, 63 patients had received oral prednisone, and 49 patients had received oral placebo. Post hoc least significant difference tests revealed serum pNF-H was reduced by 50% with IVMP with a value of 0.096 ± 0.692 ng per mL relative to placebo with a mean of 0.192 ± 0.399 ng per mL (difference = −0.067; *P* = .047) or by 40% relative to prednisone with a mean of 0.145 ± 0.134 ng per mL (difference = −0.073, *P* = .021) (Figure 1(a)). 

### 3.2. pNF-H and Clinical Parameters

Next, we investigated whether the increases in pNF-H levels of placebo and prednisone-treated patients relative to those who received IVMP, shown by the study to reduce the risk of developing MS for approximately 2 years [21], were due to optic neuritis itself or the coexistence of MS. For this analysis, we separated treatment groups into those with clinically isolated optic neuritis and those with multiple sclerosis (Figure 1(b)). Among these 175 patients, 17 (10%) initially had clinically definite multiple sclerosis (CDMS). During ONTT follow-up, another 78 (46%) developed CDMS after an average of 4.1 years. We found no significant difference (*P* = .74, ANOVA) in average pNF-H concentration between patients with onset of CDMS at or prior to the year-five visit (mean ± SD = 0.16 ± 0.31), patients with CDMS after the five-year visit (0.11 ± 0.11), or optic neuritis patients who never developed CDMS (mean ± SD = 0.14 ± 0.16). Neither was pNF-H concentration predictive of onset of CDMS after five years among the 98 patients who had not previously developed it (*P* = .51, Cox proportional hazards regression. Risk Ratio = 0.86, 95% CI: 0.54, 1.36 for a 0.1 unit higher concentration of pNF-H). Thus, reductions in pNF-H by IVMP were not due to suppression of MS, but likely due to suppression of axonal injury in the optic nerve. 

**Figure 2:**
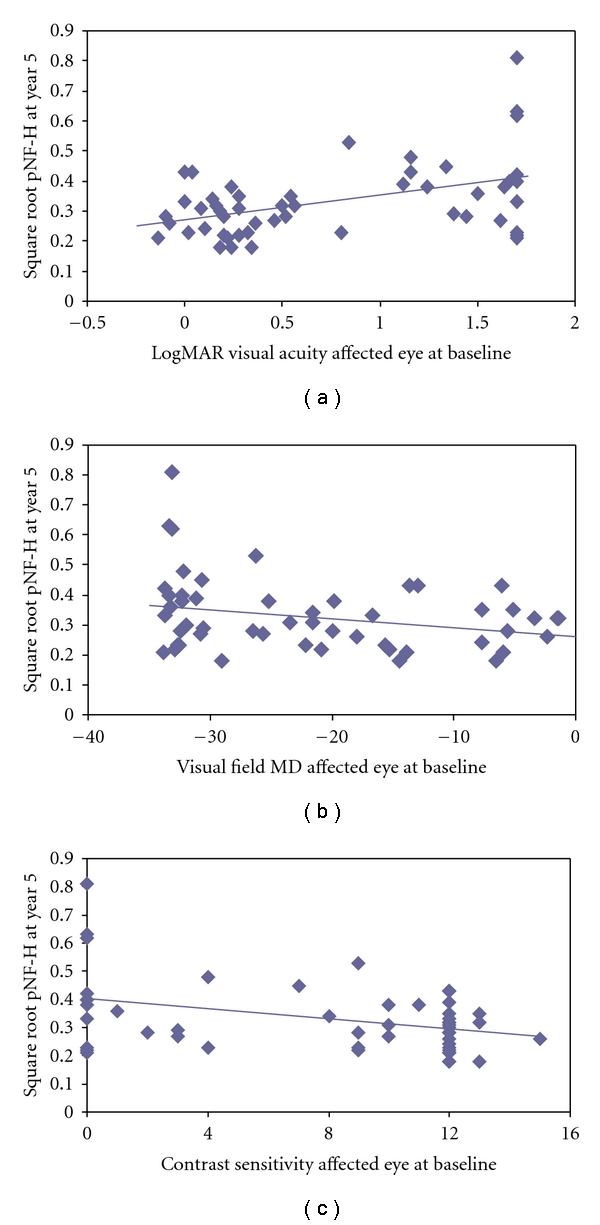
(a) A scatterplot shows the increasing pNF-H with worsening baseline visual acuity in the affected eyes with acute optic neuritis. (b) The scatterplot shows elevated serum pNF-H correlates with poorer contrast sensitivity at entry into the ONTT. (c) A scatterplot shows that higher pNF-H correlates with more severe visual field defects at baseline.

Next, we compared baseline and 5-year measurements by randomized treatment assignment and 15-year follow-up with OCT (Table 2). For the subset of 51 patients that had underwent OCTs during the year-15 visit we found that higher pNF-H levels at year 5 correlated to poorer visual acuity (*P* = .001), worse contrast sensitivity (*P* = .007), and denser visual field defects (*P* = .038) at the baseline (BL) study entry visit.  Figure 2(a) illustrates the increase in serum pNF-H with worsening baseline visual acuity in the affected eyes with acute optic neuritis.  Figure 2(b) shows that higher pNF-H correlates with more severe visual field defects at baseline.  Figure 2(c) shows higher serum pNF-H that correlates with loss of contrast sensitivity at entry into the ONTT. No correlations of visual function to pNF-H were seen in any of the follow-up visits. No correlations of pNF-H to EDSS scores only done at year 5, year ten, and year 15 were detected. No correlations of pNF-H to MRI lesions were detected.  Table 3 shows the progression of MS EDSS from year 5 to 15 for those diagnosed with MS at baseline, 6 months, 5 years, 10 years, or 15 years after study entry. Interestingly, the incidence of diagnosed MS was significantly lower in the cases that returned for the 15-year follow-up for OCT (*P* = .012). We discuss their OCT findings next. 

### 3.3. OCT

OCT signal strengths ranged from 5 to 10 in both fellow and affected eyes, with 44 (86%) and 47 (92%) having signal strength greater or equal to 7.  Table 4 shows the RNFL and macular volumes by treatment group. Differences between affected eyes relative to the fellow eyes were statistically significant.

### 3.4. OCT and Clinical Parameters

We found a significant correlation of baseline visual acuity loss to a reduction in total macular volume in the subset of 51 patients that had their RNFL and macular volumes thickness measured with Stratus OCT at the 15-year follow-up visit.  Table 5 shows the OCT correlations to visual function and EDSS for each visit alongside the pNF-H measurements. Macular volume and RNFL loss correlated to a significant loss of visual acuity at the one-month follow-up visit. Contrast sensitivity loss of affected eyes (CSENAF) and denser visual field defects (MDSE) correlated to the OCT parameters of axonal loss at almost every time point except the baseline clinical examination. 

**Figure 3:**
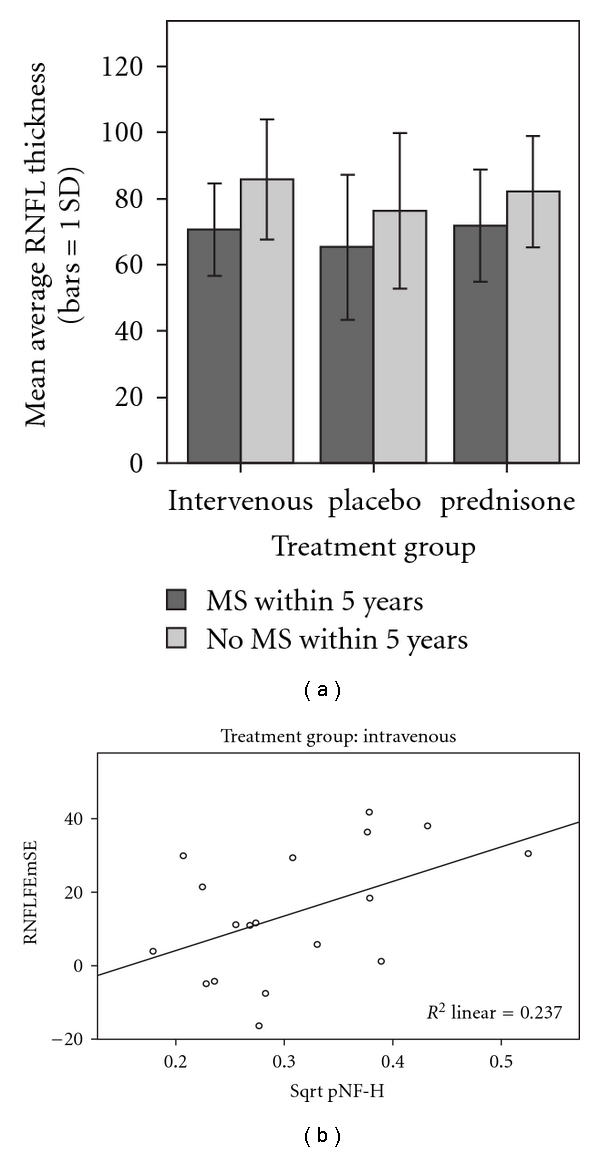
(a) A barplot of OCT RNFL thickness at 15 years in a subset of 51 patients shows no significant difference of mean RNFL with treatment of optic neuritis patients with MS or clinically isolated optic neuritis patients without MS. (b) A scatterplot of RNFL thickness difference of the fellow eye (FE) minus the study eye (SE) RNFLFEmSE correlated to serum pNF-H levels in the IVMP group.

Looking at the OCT findings by treatment group, we found no significant difference of mean IVMP RNFL = 81 *μ*m (*n* = 18), 78 *μ*m with prednisone (*n* = 18), or 71 mm placebo (*n* = 15) (Figure 3(a)). RNFL measurements of affected and nonaffected eyes of patients with CDMS (*n* = 20), respectively, 69 *μ*m and 82 *μ*m, were significantly different (resp., *P* = .018 and *P* = .001) from those with clinically isolated optic neuritis, respectively, 83 *μ*m and 99 *μ*m (*n* = 31). The average ± SD RNFL thickness in all affected eyes of 77.1 ± 19.3 *μ*m was significantly thinner than the fellow eye (*P* < .001, *t*-test) with an average 92.6 ± 17.8 *μ*m. These differences were also true for the macular volumes. 

Lastly, we found no statistical correlation of pNF-H levels to recovery of visual acuity at 6 months or RNFL thickness at 15 years in patients with or without MS. However, RNFL thickness differences of fellow eye (FE) minus study eye (SE) RNFLFEmSE correlated to pNF-H levels in the IV methylprednisolone group (*P* = .04) (Figure 3(b)). Thus, IVMP-treated patients with greater loss of axons at year 15 had increased pNF-H levels at year 5.

## 4. Discussion

We have shown here that serum pNF-H levels measured 5 years after acute optic neuritis correlate with baseline visual function. Next, early follow-up visual function correlates with RNFL and macular volumes measured at 15 years. Lastly, pNF-H levels at year 5 do not correlate with RNFL and macular volumes in the affected eye, but they do correlate with the difference between study and fellow eyes at least in the IVMP group. 

Our findings of increased pNF-H levels with more severe baseline visual deficits are consistent with axonal transection by inflammatory cells that is believed to contribute to the loss of axons [8]. This insult occurs at baseline, and the axons start the process of dying off, releasing pNF-H into the serum as they do. This process of degeneration appears to continue for at least 5 years. OCT measures what is remaining after the axons have died off, thus a better correlation to the follow-up exams. Teunissen et al. showed that neurofilament heavy chain levels detected in the CSF of patients with MS and clinically isolated syndrome were higher than normal controls, reaching their highest levels during acute exacerbations of disease activity [22]. Petzold et al., who obtained serum samples for measurements of the pNF-H during the acute phases of optic neuritis, found higher levels in those who had a poor visual recovery [12]. Since acute serum samples from ONTT patients were unavailable for inclusion in our study here, we had only a single snapshot of pNF-H, at year 5. Therefore, we are unable to resolve whether axonal transection by inflammatory cells was suppressed by IVMP or whether treatment induced a decrease in release of pNF-H. Still, due to its high resistance to protease digestion [9], pNF-H may have persisted at higher levels for years, though this seems unlikely. Alternately, a small amount of axonal loss and regeneration may be an ongoing feature of optic neuropathy, and IVMP may have a long-term effect by reducing this.

While we found no correlation of pNF-H to clinical measures of visual function at the very late time points, and pNF-H was not associated with study eye RNFL even in the IVMP group, the difference between fellow eye and study eye RNFL thickness correlated with pNF-H levels in the IVMP group. This finding validates pNF-H as a biomarker of suppression of axonal loss by high-dose methylprednisolone, now standard therapy for acute optic neuritis and MS exacerbations. Since no difference in serum pNF-H concentration between clinically isolated optic neuritis and MS patients were detected, it is unlikely that brain lesions outside the optic nerve contributed to the differences in pNF-H between the IV methylprednisolone and the other two treatment groups. We also excluded brain lesions in clinically isolated optic neuritis and multiple sclerosis patients detected on T2-weighted MRI as a cause for the differences in pNF-H between the treatment groups. 

Consistent with our observations in ONTT patients, elevation of serum pNF-H has also been found in another disease that affects the optic nerve, Lebers' hereditary optic neuropathy (LHON) [23]. Elevations in serum pNF-H have also been detected in animals with experimental autoimmune encephalomyelitis (EAE) and suppressed with treatment [24]. As further verification that axonal loss from optic neuritis was the likely source of the pNF-H in ONTT patients, we turned to transgenic TCR MOG mice that develop only optic neuritis that in turn causes loss of retinal ganglion cells and their optic nerve axons [25]. Preliminary unpublished observations in these animals revealed a several fold elevation in serum pNF-H levels relative to control littermates (presented at the AAN Toronto 2010—[P06.229]). Thus, serum pNF-H elevations in ONTT patients were likely due to optic neuritis, and this biomarker is likely a reliable monitor for suppression of axonal injury in the optic nerve. 

Despite the OCT and pNF-H correlation data supporting a long-term benefit of treatment with IVMP, its effect on visual parameters and neurologic or MRI lesions was virtually nil. For month 1, month 6, year 5, year 10, and year 15, treatment had very little affect on outcome variables of acuity, contrast sensitivity, visual field mean deviation, EDSS score, or MRI lesions [1]. Only contrast sensitivity at month 1 and month 6, and mean deviation at month 6 were, or approached being, significantly different with IVMP. Thus, contrast sensitivity may be a sensitive clinical measure of treatment outcome. In fact, using low contrast acuity as an outcome measure, Balcer and colleagues demonstrated that natalizumab treatment of MS patients was beneficial to this parameter of visual function only [26]. That study did not examine the effects of treatment on axonal or neuronal loss. 

Corticosteroids have been shown to suppress axonal loss in the optic nerves of animals with experimental autoimmune encephalomyelitis (EAE) [27]. On the other hand, in this animal model of MS, corticosteroids have also been shown to induce neuronal degeneration in retinal ganglion cells whose axons comprise the optic nerve [28]. Thus, corticosteroids appear to have opposing effects on different elements of the visual system in animal experimental models. In optic neuritis associated with neuromyelitis optica (NMO), IVMP treatment resulted in a better visual outcome and suppressed RNFL loss [29]. In a study of MS, no differences in RNFL were detected between patients treated with interferon beta-1a, interferon beta-1b, or glatiramer acetate relative to placebo [30]. To our knowledge, our study is the first demonstrating that an anti-inflammatory pharmacologic agent is able to modulate a marker of axonal loss in optic neuritis patients that do not have NMO. Since axonal loss is a characteristic of optic neuritis that may be slightly suppressed by methylprednisolone at a dose of 1 gram a day for 3 days, one can only wonder whether substantial escalation of the dose would have a greater neuroprotective effect than that seen here. Perhaps, those patients with worse baseline visual function found to have elevated pNF-H could be targeted with a neuroprotective strategy [31] as pNF-H levels too correlated with loss of RNFL and macular volumes ten years later.

## Figures and Tables

**Table 1:** Baseline (recruitment) participant characteristics of ONTT subjects undergoing pNF-H testing.

Number	175
Mean (SD) [range] age	33.2 (6.8) [18,46]
*N* (%) Female	140 (80%)
*N* (%) Race/Ethnicity	
Caucasian	159 (91%)
African-American	15 (9%)
Hispanic	1 (1%)
*N* (%) Right eye affected	92 (53%)
*N* (%) Initial multiple sclerosis	
None	114 (65%)
Possible	35 (20%)
Probable	9 (5%)
Definite	17 (10%)
Treatment group	
IVMP	63 (36%)
Prednisone	63 (36%)
Placebo	49 (28%)

**Table 2:** Visual and clinical scores by treatment group and OCT.

Treatment group	IVMP		Placebo		Prednisone		Total		Total
OCT at 15 yrs	Yes	No	Yes	No	Yes	No	Yes	No	Total
*N*	18	45	15	34	18	45	51	124	175

Mean ± SD LogMAR acuity									
Affected eye									
Baseline	0.78 ± 0.65	0.66 ± 0.63	0.59 ± 0.71	0.67 ± 0.72	0.86 ± 0.67	0.86 ± 0.69	0.75 ± 0.67	0.74 ± 0.68	0.74 ± 0.68
Year 5	−0.08 ± 0.14	−0.06 ± 0.15	−0.05 ± 0.11	−0.02 ± 0.37	−0.07 ± 0.16	0.01 ± 0.32	−0.07 ± 0.14	−0.03 ± 0.29	−0.04 ± 0.25
Year 10	−0.08 ± 0.10	0.01 ± 0.35	−0.03 ± 0.09	−0.07 ± 0.25	−0.07 ± 0.11	0.06 ± 0.37	−0.06 ± 0.10	0.01 ± 0.33	−0.01 ± 0.28
Year 15	−0.07 ± 0.11	0.06 ± 0.38	−0.02 ± 0.10	0.04 ± 0.28	−0.06 ± 0.10	0.11 ± 0.36	−0.05 ± 0.11	0.07 ± 0.34	0.03 ± 0.29
Fellow eye									
Baseline	−0.12 ± 0.07	−0.12 ± 0.09	−0.13 ± 0.10	−0.08 ± 0.22	−0.14 ± 0.08	−0.14 ± 0.09	−0.13 ± 0.08	−0.12 ± 0.14	−0.12 ± 0.12
Year 5	−0.14 ± 0.14	−0.12 ± 0.11	−0.11 ± 0.10	−0.08 ± 0.32	−0.17 ± 0.09	−0.07 ± 0.30	−0.14 ± 0.12	−0.09 ± 0.26	−0.11 ± 0.22
Year 10	−0.10 ± 0.15	−0.07 ± 0.21	−0.07 ± 0.11	−0.15 ± 0.09	−0.15 ± 0.08	−0.05 ± 0.29	−0.11 ± 0.12	−0.08 ± 0.22	−0.09 ± 0.19
Year 15	−0.10 ± 0.15	0.01 ± 0.30	−0.09 ± 0.09	−0.04 ± 0.12	−0.12 ± 0.09	−0.01 ± 0.32	−0.10 ± 0.11	−0.01 ± 0.27	−0.05 ± 0.23

Mean ± SD contrast sensitivity									
Affected eye									
Baseline	7.8 ± 4.8	8.1 ± 4.6	8.6 ± 5.6	7.6 ± 5.1	7.4 ± 5.4	7.0 ± 4.9	7.9 ± 5.2	7.5 ± 4.8	7.6 ± 4.9
Year 5	14.3 ± 1.3	14.0 ± 1.5	13.9 ± 1.5	13.8 ± 2.8	14.8 ± 0.9	13.3 ± 3.0	14.4 ± 1.3	13.7 ± 2.5	13.9 ± 2.2
Year 10	11.6 ± 0.9	11.4 ± 2.2	11.3 ± 1.3	11.4 ± 2.4	11.8 ± 1.0	10.9 ± 2.5	11.6 ± 1.1	11.2 ± 2.4	11.3 ± 2.0
Year 15	11.6 ± 1.1	11.1 ± 2.7	11.3 ± 1.3	11.2 ± 2.7	11.5 ± 1.5	10.8 ± 2.9	11.5 ± 1.3	11.0 ± 2.8	11.2 ± 2.3
Fellow eye									
Baseline	15.2 ± 0.7	15.6 ± 0.6	14.9 ± 0.6	15.0 ± 1.3	15.4 ± 0.6	15.3 ± 0.8	15.2 ± 0.7	15.3 ± 0.9	15.3 ± 0.9
Year 5	15.3 ± 1.0	15.2 ± 0.7	15.1 ± 0.5	14.8 ± 2.1	15.7 ± 0.5	14.7 ± 2.6	15.4 ± 0.7	14.9 ± 2.0	15.1 ± 1.7
Year 10	12.2 ± 1.4	12.0 ± 1.2	12.3 ± 0.5	12.2 ± 0.9	12.2 ± 0.6	11.7 ± 2.3	12.3 ± 0.9	11.9 ± 1.6	12.0 ± 1.4
Year 15	12.1 ± 1.5	11.8 ± 1.8	12.5 ± 0.8	12.1 ± 1.0	12.5 ± 0.7	11.5 ± 2.7	12.4 ± 1.1	11.8 ± 2.0	12.0 ± 1.7

Mean ± SD mean deviation									
Affected eye									
Baseline	−22.0 ± 11.3	−21.5 ± 9.9	−19.2 ± 10.6	−21.6 ± 10.0	−23.4 ± 11.2	−23.7 ± 10.2	−21.7 ± 11.0	−22.3 ± 10.0	−22.1 ± 10.3
Year 5	−2.0 ± 2.1	−1.3 ± 4.0	−5.1 ± 7.9	−3.0 ± 8.1	−1.5 ± 2.6	−3.9 ± 7.4	−2.7 ± 4.9	−2.7 ± 6.6	−2.7 ± 6.2
Year 10	−1.3 ± 2.8	−2.5 ± 5.1	−5.0 ± 7.6	−3.0 ± 6.9	−1.5 ± 1.8	−4.1 ± 7.3	−2.5 ± 4.8	−3.3 ± 6.5	−3.0 ± 6.0
Year 15	−1.0 ± 1.9	−1.7 ± 5.7	−5.1 ± 7.2	−2.9 ± 6.9	−1.6 ± 2.1	−3.8 ± 6.6	−2.4 ± 4.5	−2.8 ± 6.4	−2.7 ± 5.7
Fellow eye									
Baseline	−2.1 ± 1.9	−2.8 ± 1.9	−2.7 ± 2.3	−3.3 ± 3.5	−3.6 ± 2.5	−3.8 ± 4.6	−2.8 ± 2.3	−3.3 ± 3.5	−3.1 ± 3.2
Year 5	−1.1 ± 2.3	−0.6 ± 1.9	−1.8 ± 5.3	−1.1 ± 5.1	−0.1 ± 2.4	−2.5 ± 6.5	−1.0 ± 3.5	−1.4 ± 4.9	−1.3 ± 4.5
Year 10	−0.7 ± 2.7	−1.7 ± 4.6	−1.7 ± 4.9	−1.2 ± 2.7	0.0 ± 1.2	−2.3 ± 7.1	−0.8 ± 3.2	−1.8 ± 5.3	−1.5 ± 4.8
Year 15	−0.3 ± 2.9	−1.3 ± 5.5	−2.0 ± 5.2	−0.7 ± 2.4	−0.2 ± 1.7	−2.6 ± 5.0	−0.8 ± 3.5	−1.6 ± 4.6	−1.3 ± 4.2

*N* (%) diagnosed with multiple sclerosis									
Baseline	0	3 (7%)	1 (7%)	4 (12%)	1 (6%)	8 (18%)	2 (4%)	15 (12%)	17 (10%)
Year 5	4 (22%)	14 (31%)	3 (20%)	20 (59%)	3 (20%)	6 (33%)	13 (26%)	59 (48%)	72 (41%)
Year 10	5 (28%)	17 (38%)	5 (33%)	23 (68%)	6 (33%)	30 (67%)	16 (31%)	70 (57%)	86 (49%)
Year 15	6 (33%)	20 (44%)	7 (47%)	23 (68%)	7 (39%)	32 (71%)	20 (39%)	75 (61%)	95 (54%)

**Table 3:** MS characteristics.

	Diagnosis of MS					All
	Baseline, *N* = 17	Month 6, *N* = 11	Year 5, *N* = 44	Year 10, *N* = 14	Year 15, *N* = 9	*N* = 95
Mean (SD) EDSS						
Year 5	1.97 (1.59)	1.77 (1.82)	1.30 (0.98)	0.71 (1.09)*	0.22 (0.44)**	1.28 (1.29)
Year 10	2.79 (1.88)	1.96 (1.96)	2.42 (2.41)	2.35 (2.13)	0.78 (0.79)***	2.24 (2.14)
Year 15	3.83 (1.90)	2.55 (1.62)	2.94 (2.46)	2.96 (2.12)	1.33 (1.36)	2.84 (2.18)
Year 5 mean (SD) sqrt pNFH						
IV	0.29 (0.04)	0.24 (0.11)	0.25 (0.11)	0.33 (0.09)	0.22 (0.05)	0.26 (0.09)
Prednisone	0.45 (0.13)	0.36 (0.20)	0.33 (0.16)	0.31 (0.07)	0.41 (0.36)	0.37 (0.17)
Placebo	0.20 (0.13)	0.21 (0.20)	0.46 (0.34)	0.30 (0.06)	0.34 (0.11)	0.36 (0.27)
	*P* = .008	*P* = .52	*P* = .065	*P* = .85	*P* = .52	*P* = .041
≥5 MRI lesions at baseline	13 (77%)	3 (30%)	16 (42%)	4 (29%)	4 (57%)	40 (47%)
Year 15 mean (SD) RNFL	*N* = 2	*N* = 3	*N* = 8	*N* = 3	*N* = 4	*N* = 20
Affected eye	79.4 (4.6)	70.0 (8.1)	69.7 (19.8)	65.0 (17.0)	66.0 (25.5)	69.3 (17.4)
Fellow eye	94.6 (3.8)	74.2 (16.3)	82.0 (19.0)	97.3 (2.7)	73.1 (24.5)	82.6 (18.3)
Difference	15.2 (0.8)	4.2 (8.5)	12.4 (16.4)	32.3 (15.1)	7.1 (6.5)	13.3 (14.7)
Year 15 mean (SD) macular volume						
Affected eye	7.1 (0.9)	6.1 (0.7)	6.0 (0.5)	6.8 (0.5)	6.2 (0.6)	6.3 (0.6)
Fellow eye	7.6 (0.1)	6.2 (0.8)	6.6 (0.6)	7.3 (0.9)	6.7 (0.7)	6.7 (0.7)
Difference	0.5 (0.8)	0.1 (0.2)	0.6 (0.5)	0.5 (0.4)	0.2 (0.2)	0.4 (0.4)

**Table 4:** OCT data RNFL and macular volumes.

	IVMP *N* = 18	Placebo *N* = 15	Prednisone *N* = 18	Total *N* = 51
Signal strength				
Affected eye	8.2 ± 1.2	8.2 ± 1.2	7.9 ± 1.5	8.1 ± 1.3
Fellow eye	8.1 ± 1.8	8.5 ± 1.1	8.4 ± 1.2	8.3 ± 1.4
Year 15 mean (SD) RNFL				
Affected eye	80.9 ± 18.1	71.3 ± 22.7	78.2 ± 17.1	77.1 ± 19.3
Fellow eye	95.2 ± 16.7	87.1 ± 18.1	94.5 ± 18.5	92.6 ± 17.8
Difference (AF-FE)	−14.3 ± 17.3	−15.8 ± 22.4	−16.3 ± 15.9	−15.5 ± 18.2
*P*-value	.003	.016	<.001	<.001
Year 15 mean (SD) macular volume				
Affected eye	6.3 ± 0.5	6.5 ± 0.7	6.4 ± 0.5	6.4 ± 0.6
Fellow eye	6.7 ± 0.5	6.9 ± 0.8	7.0 ± 0.6	6.9 ± 0.7
Difference	−0.4 ± 0.5	−0.4 ± 0.5	−0.7 ± 0.6	−0.5 ± 0.6
*P*-value	.005	.010	<.001	<.001

**Table 5:** Correlations of clinical profile to OCT and pNF-H.

Measurement	Visit	Statistic	Study eye RNFL thickness	Study eye total macular volume	Square root pNFH
EDSS	Year 5	*r*	−0.28	−0.28	0.15
		*P*-value	.048	.057	.291
		*N*	50	47	50
	Year 10	*r*	−0.38	−0.11	0.02
		*P*-value	.008	.473	.901
		*N*	49	46	49
	Year 15	*r*	−0.23	0.07	0.03
		*P*-value	.107	.641	.841
		*N*	51	48	51

LogMAR study eye acuity	Baseline	*r*	−0.09	−0.29	0.44
		*P*-value	.512	.046	.001
		*N*	51	48	51
	Month 1	*r*	−0.44	−0.41	0.13
		*P*-value	.002	.005	.364
		*N*	48	45	48
	Month 6	*r*	−0.35	−0.22	−0.11
		*P*-value	.011	.132	.442
		*N*	51	48	51
	Year 5	*r*	−0.27	−0.26	−0.18
		*P*-value	.060	.074	.206
		*N*	51	48	51
	Year 10	*r*	−0.52	−0.30	−0.08
		*P*-value	<.001	.040	.565
		*N*	50	47	50
	Year 15	*r*	−0.48	−0.36	0.00
		*P*-value	<.001	.011	.974
		*N*	51	48	51

Sensitivity study eye contrast	Baseline	*r*	0.18	0.23	−0.37
		*P*-value	.207	.121	.007
		*N*	51	48	51
	Month 1	*r*	0.54	0.42	−0.17
		*P*-value	<.001	.004	.256
		*N*	48	45	48
	Month 6	*r*	0.54	0.44	−0.12
		*P*-value	<.001	.002	.419
		*N*	51	48	51
	Year 5	*r*	0.45	0.37	−0.04
		*P*-value	.001	.010	.759
		*N*	50	47	50
	Year 10	*r*	0.52	0.43	0.01
		*P*-value	<.001	.003	.964
		*N*	49	46	49
	Year 15	*r*	0.58	0.52	−0.06
		*P*-value	<.001	<.001	.685
		*N*	51	48	51
Study eye visual field mean deviation	Baseline	*r*	0.24	0.30	−0.29
		*P*-value	.095	.036	.038
		*N*	51	48	51
	Month 1	*r*	0.56	0.46	−0.11
		*P*-value	<.001	.001	.462
		*N*	48	45	48
	Month 6	*r*	0.55	0.34	0.08
		*P*-value	<.001	.019	.593
		*N*	51	48	51
	Year 5	*r*	0.48	0.32	0.06
		*P*-value	<.001	.029	.650
		*N*	51	48	51
	Year 10	*r*	0.57	0.40	0.10
		*P*-value	<.001	.006	.512
		*N*	50	47	50
	Year 15	*r*	0.55	0.30	0.05
		*P*-value	<.001	.041	.730
		*N*	51	48	51
